# FATP4 missense and nonsense mutations cause similar features in Ichthyosis Prematurity Syndrome

**DOI:** 10.1186/1756-0500-4-90

**Published:** 2011-03-30

**Authors:** Maria Sobol, Niklas Dahl, Joakim Klar

**Affiliations:** 1 Department of Immunology, Genetics and Pathology, Science for Life Laboratory and Rudbeck Laboratory, Uppsala University, Uppsala, Sweden; 2 Department of General and Molecular Genetics, National Taras Shevchenko University of Kyiv, Kyiv, Ukraine

## Abstract

**Background:**

Ichthyosis Prematurity Syndrome (IPS) is an autosomal recessive disorder characterized by premature birth, non-scaly ichthyosis and atopic manifestations. The disease was recently shown to be caused by mutations in the gene encoding the fatty acid transport protein 4 (FATP4) and a specific reduction in the incorporation of very long chain fatty acids (VLCFA) into cellular lipids.

**Findings:**

We screened probands from five families segregating IPS for mutations in the *FATP4 *gene. Four probands were compound heterozygous for four different mutations of which three are novel. Four patients were heterozygous and one patient homozygous for the previously reported non-sense mutation p.C168X (c.504c > a). All patients had clinical characteristics of IPS and a similar clinical course.

**Conclusions:**

Missense mutations and non-sense mutations in *FATP4 *are associated with similar clinical features suggesting that missense mutations have a severe impact on FATP4 function. The results broaden the mutational spectrum in *FATP4 *associated with IPS for molecular diagnosis of and further functional analysis of FATP4.

## Introduction

Ichthyosis prematurity syndrome (IPS) is a rare form of autosomal recessive ichthyosis characterized by polyhydramnion and premature birth of the affected child [1-3]. Newborns exhibit respiratory complications and a thick caseous desquamating epidermis. During the first months of life the symptoms become gradually milder. Patients suffer from a lifelong non-scaly ichthyosis with atopic manifestations. Ultrastructural analyses reveal membrane aggregations in the upper epidermal layers, and histological analysis of the skin reveal thickening of the epidermis. Patients with IPS have been reported from Scandinavia, Middle East, southern Europe and Africa [2,4,5]. However, IPS is more prevalent in Norway and Sweden with an estimated local carrier frequency of one in 50 suggesting a founder mutation [1].

Linkage and haplotype analysis followed by candidate gene sequencing of IPS patients have shown that the disease is associated with mutations in the fatty acid transport protein 4 (*FATP4*) gene [2]. The FATP4 protein plays a central role in the transport and activation of fatty acids in the epidermis and for normal epidermal barrier function [6,7]. The *FATP4 *gene consists of 13 exons encoding a peptide with a predicted size of 72 kDa spanning a N-terminal transmembrane (TM) domain, an endoplasmatic reticulum localization signal (ERx) domain, ATP/AMP and FATP motifs of AMP-binding domain [6]. The FATP domains are involved in binding and uptake of long chain fatty acids (LCFA) and very long chain fatty acids (VLCFA) as well as catalysis and esterification of fatty acids in the presence of CoA [6-9].

So far, eight distinct *FATP4 *mutations have been reported in IPS patients. One nonsense mutation, p.C168X, five missense mutations p.A92T, p.S247P, p.Q300R, p.R374C and p.R583 H, and two splice site mutations, c.716-1ag > aa and c.988-2ag > gg [2,10]. The p.C168X nonsense variant is prevalent in the Scandinavian IPS population and all patients from this geographical region were found homozygous or compound heterozygous for this mutation. In order to clarify the mutation spectrum in IPS we screened five non-related probands of Scandinavian descent for novel *FATP4 *mutations. We report herein on the identification of *FATP4 *mutations in these probands including three novel mutations. All six patients were compound heterozygous or homozygous for the p.C168X mutation which further illustrates the high prevalence for this mutation in the northern European population.

## Methods

### Patient material

**Figure 1:**
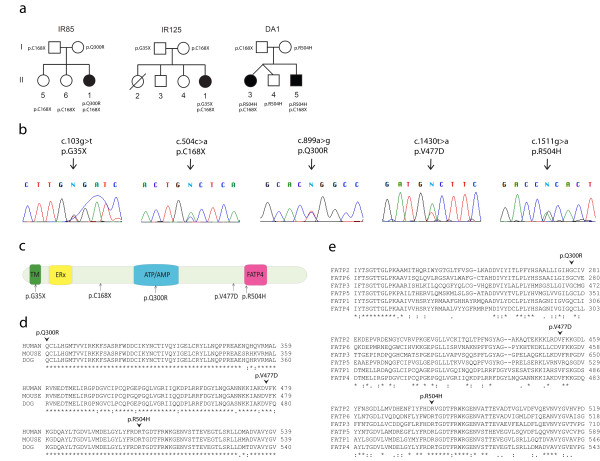
**Family pedigrees, mutation traces and positions of predicted amino acid substitutions in FATP4**. Multiple sequence alignment is shown of FATP4 protein with orthologs from different species and with the FATP family of proteins. (a) Pedigrees of three of the five families from which healthy family members were available showing the segregation of mutations and IPS (b) Sequence chromatograms of the five mutations identified (c) Schematic overview of FATP4 functional domains; the N-terminal transmembrane region (TM), the ER localization signal (ERx; aa 47-102), the ATP/AMP motif involved in ATP binding and adenylate formation (ATP/AMP; aa 243-345) and the conserved FATP motif of importance for fatty acid binding (FATP; aa 500-551). The arrows indicate positions of the mutated amino acid residues reported herein associated with IPS (d) Multiple sequence alignment of selected regions of the human FATP4 protein and Fatp4 proteins from mouse and dog. Arrows indicate the position of the missense mutations at conserved residues (e) Multiple sequence alignment of FATP4 protein with the human FATP family of proteins (FATP1, FATP2, FATP3, FATP5, and FATP6). The positions of the missense mutations are indicated by arrows

We analyzed six cases with a typical IPS phenotype. The patients belongs to five families from Norway (IR85, IR125, and 25187), Denmark (DA1 comprising two affected siblings) and Iceland (25291; Table 1). No consanguinity was reported among parents and no families were inter-connected through the last four generations. The patients had a medical history with the clinical triad of premature birth, thick caseous desquamating epidermis, and neonathal asphyxia, typical for the IPS phenotype [11]. With the exception of Family DA1 (Figure 1a), no family history of IPS was recorded. Blood samples were obtained from probands and from healthy parents and siblings in three families (Family DA1, IR85 and IR125; Figure 1a).

**Table 1:** Mutations in the *FATP4 *gene identified in probands from five IPS families.

Patient family ID	Exon	Nucleotide change	Amino-acid change	Zygosity	Origin
**IR85**	exon3 exon7	c.504c>a c.899a > g	p.C168X* p.Q300R*	compound heterozygous	Norway
**IR125**	exon2 exon3	c.103g>t c.504c > a	p.G35X p.C168X*	compound heterozygous	Norway
**25187**	exon3 exon10	c.504c>a c.1430t > a	p.C168X* p.V477D	compound heterozygous	Norway
**25291**	exon3	c.504c > a	p.C168X*	homozygous	Iceland
**DA1**	exon3 exon11	c.504c>a c.1511 g > a	p.C168X* p.R504H	compound heterozygous	Denmark

Family origin and zygosity of the identified mutations. The nucleotide and amino-acid change is indicated and the corresponding exon. *denotes previously published in Klar et al 2009.

### Mutational analysis

Sequence analysis of the *FATP4 *gene was initially performed on the probands. In families DA1, IR85 and IR125, available parents and siblings were included for the investigation of mutational inheritance (Figure 1a). In total, 16 individuals (six patients and ten relatives) were included in the mutation analysis. DNA samples from 100 unrelated healthy Scandinavian control individuals were also analyzed to rule out polymorphic variants. Bidirectional sequence analysis of genomic DNA included all 13 exons of the *FATP4 *gene as well as intron-exon boundaries, 5' and 3' UTRs. Primer sequences used for amplification were designed with Primer 3 software (primer sequences available upon request). Sequencing was performed on an ABI 3730 DNA Analyzer (Applied Biosystems) using Big Dye Terminator v3.1 Cycle Sequencing Chemistry (Applied Biosystems) according to protocols recommended by the manufacturer. Base calling was performed with Sequencing Analysis v 5.2 (Applied Biosystems) and the sequences were analyzed with the Sequencher software v4.1 (Gene Codes Corporation).

### Structural modelling

To analyze the degree of conservation of the predicted amino acid substitutions, multiple sequence alignment was performed using ClustalW [12]. The human (*Homo sapiens*) FATP4 protein sequence was compared to mouse (*Mus musculus*) and dog (*Canis familiaris*) as well as the other members of the FATP protein family (FATP1, FATP2, FATP3, FATP5 and FATP6)

## Results

### Mutational analysis

Sequence analysis of the *FATP4 *gene revealed two sequence variants predicted to be associated with IPS in each proband. In total five different mutations were identified (Table 1). Five probands were compound heterozygous for the previously reported c.504c > a transition in exon 3. This variant predicts the previously reported p.C168X nonsense mutation. One proband from the Icelandic family 25291 was found homozygous for this mutation. We also identified a novel c.103g > t transition in exon 2. This variant results in a p.G35X nonsense mutation (Figure 1b) and an early truncation of the protein. Another three sequence variants were predicted to result in missense mutations. The p.Q300R is caused by an a to g transition at nucleotide 899 (c.899g > a) in exon 7 and results in the substitution of the polar uncharged hydrophilic amino acid glutamine for the positively charged hydrophilic arginine. This mutation has been reported previously [2]. The novel p.V477 D is caused by a t to a transition at nucleotide 1430 (c.1430t > a) in exon 10 and results in the change of the nonpolar hydrophobic amino acid valine to the negatively charged hydrophilic aspartic acid. The p.R504 H mutation is another novel variant caused by a g to a transition at nucleotide 1511 (c.1511g > t) in exon 11 and results in the substitution of the positively charged hydrophilic arginine for the similarly positively charged hydrophilic histidine (Table 1). Healthy family members were available in family IR85, IR125 and DA1 and the analysis showed that the mutations segregated with the disease with parents being heterozygous carriers for the respective mutations (Figure 1a). The three missense mutations (p.Q300R, p.V477 D and p.R504H) were situated in the highly conserved AMP-binding domain (aa 103-536). The p.Q300R is situated in the AMP/AMP motif involved in ATP binding and adenylate formation, and the novel p.R504 H is located in the FATP motif important for fatty acid binding (Figure 1c). All variants were excluded as common variants or polymorphisms by comparing to databases (dbSNP 131) and by the analysis of 200 Scandinavian control chromosomes. Thus, among five different IPS associated mutations identified in the five families, the p.G35X, p.V477 D and p.R504 H mutations are novel whereas the two mutations (p.C168X and p.Q300R) were described previously [2].

### Structural modelling

Multiple sequence alignment of the FATP4 protein was performed in order to estimate inter-species conservation of certain segments and to identify regions shared with other members of the FATP family of proteins. Alignment of the FATP4 protein sequence from human (*Homo sapiens*), mouse (*Mus musculus*) and dog (*Canis familiaris*) demonstrated strong conservation of the three amino acids corresponding to the missense mutations in the patients (Figure 1d). Alignment of the FATP4 sequence to other members of the FATP family of proteins showed complete identity of the amino acids corresponding to the novel missense mutations, p.V477 and p.R504 (Figure 1e). The previously identified missense mutation, p.Q300R, is situated in a protein domain shared exclusively with FATP1.

## Discussion

In this study we report on five families from Scandinavia with members affected by IPS. Sequence analysis revealed five different mutations, of which three are novel and two (p.C168X and p.Q300R) have been reported previously [2]. All patients were either homozygous or compound heterozygous for the nonsense mutation p.C168X, confirming this to be a prevalent ancestral mutation in the Scandinavian population. The novel variants include one nonsense (p.G35X) and two novel missense mutations (p.V477 D and p.R504H) in our cohort of patients. Both missense mutations are situated in the highly conserved AMP binding domain of the FATP4 protein supporting their functional significance. The p.R504 H mutation is also the first mutation described in the FATP motif within this domain. The functional significance of this mutation is supported by studies of the FATP1 member of the FATP family of proteins demonstrating that mutations in the FATP motif results in an almost complete elimination of both transport and activation of fatty acids [13].

## Conclusions

With the exception of the ancestral p.C168X mutation, the IPS associated mutational spectrum in FATP4 appear heterogeneous and with a predilection to functional FATP domains. However, from our observations there appear to be no obvious correlations between the nature of mutations in the FATP4 protein and the IPS phenotype. This suggests that the missense mutations reported herein have deleterious effects on FATP4 function which are similar to the effects of the nonsense mutations. Our findings also confirm the critical role of FATP4 for the development and maintenance of the skin barrier. Furthermore, this study adds to the mutational spectrum associated with IPS, which may improve genetic diagnosis of the disease as well as future functional analysis of FATP4.

## Competing interests

None of the authors have a commercial or other association that might pose a conflict of interest.

## Authors' contributions

MS carried out the genetic studies and helped to draft the manuscript. ND participated in the study design and helped to draft the manuscript. JK designed the study, interpreted the data and drafted the manuscript. All authors read and approved the final manuscript.
